# A Novel 3D-Printed Device for Precise Percutaneous Placement of Cannulated Compression Screws in Human Femoral Neck Fractures

**DOI:** 10.1155/2021/1308805

**Published:** 2021-06-10

**Authors:** Cheng Long, Jin-hai Liu, Xiang-ping Chai, Xiang-feng Liu, Zhi-xi Duan

**Affiliations:** ^1^Department of Orthopedics, Xiangya Hospital, Central South University, No. 87 Xiangya Road, Changsha 410008, China; ^2^Department of Orthopedics, Qingdao Chengyang Guzhen Orthopedic Hospital, No. 518 Yinhe Road, Chengyang District, Qingdao 266107, China; ^3^Department of Emergency Medicine, Trauma Center, The Second Xiangya Hospital, Central South University, No. 139 Renmin Road, Changsha, 410011 Hunan, China

## Abstract

**Background:**

The aim of this study was to investigate the application of computer-aided design and 3D printing technology for percutaneous fixation of femoral neck fractures using cannulated compression screws.

**Methods:**

Using computed tomography data, an individualized proximal femur model was created with a 3D printer. The reduction of the femoral neck fracture and the placement of the cannulated compression screws were simulated on a computer. A 3D printing guide plate was designed to match the proximal femur. After demonstrating the feasibility of the 3D model before the surgical procedure, the guide needles and cannulated compression screws were inserted with the aid of the 3D-printed guide plate.

**Results:**

During the procedure, the 3D-printed guide plate for each patient matched the bone markers of the proximal femur. With the aid of the 3D-printed guide plate, three cannulated compression screws were accurately inserted into the femoral neck to treat femoral neck fractures. After screw placement, intraoperative X-ray examination showed results that were consistent with the preoperative design. The time taken to complete the procedure in the guide plate group was 35.3 ± 2.1 min, the intraoperative blood loss was 6.3 ± 2.8 mL, and X-ray fluoroscopy was only performed 9.1 ± 3.5 times. Postoperative radiographs showed adequate reduction of the femoral neck fractures. The entry point, entry direction, and length of the three cannulated compression screws were consistent with the preoperative design, and the screws did not penetrate the bone cortex.

**Conclusion:**

Using computer-aided design and 3D printing technology, personalized and accurate placement of cannulated compression screws can be realized for the treatment of femoral neck fractures. This technique can shorten the time required for the procedure and reduce damage to the femoral neck cortex, intraoperative bleeding, and the exposure of patients and healthcare staff to radiation.

## 1. Introduction

A femoral neck fracture is the third most common type of fracture in trauma orthopedics, and it accounts for approximately 41% of hip fractures and 3.6% of total fractures [1–3]. There are approximately 1.5 million hip fractures every year globally, and with the aging of the population, this is expected to increase to 6.3 million by 2050 [4]. It is estimated that approximately 30% of hip fractures in the world occur in Asian countries, especially in China [5].

The femoral neck fracture often occurs in elderly patients with osteoporosis, mainly due to low-energy injury [6]. Its incidence is very low in young patients, in whom it occurs mainly due to high-energy trauma. Despite surgical interventions, the reoperation rates for and mortality after this type of fracture remain high [7–9]. Although there are many methods to treat femoral neck fractures, cannulated compression screw fixation remains the mainstream surgical intervention [10–12].

In recent years, computer-aided design and 3D printing technology have become more widely used in the medical field [13]. In particular, considerable progress has been made in the field of orthopedics. At present, these techniques are mainly used for preoperative planning, the 3D printing of guide plates, and personalized implant production [14–16]. 3D-printed guide plates are usually used to guide surgeons to accurately insert internal fixation screws and plates. In this study, we used computer-aided design and 3D printing technology to develop a new type of guide plate for the precise and rapid percutaneous fixation of femoral neck fractures.

## 2. Materials and Methods

### 2.1. Patient Information

From February 2019 to February 2020, 40 patients with a femoral neck fracture were operated in our hospital, and they were divided into two groups according to their preferences. Patients were included in the study if they presented with a simple femoral neck fracture without neurovascular injury. They were treated with three cannulated compression screws. Forty patients with femoral neck fractures were included in the study. Twenty patients were treated with 3D-printed guide plate-assisted screw placement (guide plate group), and the remaining 20 were treated with freehand screw placement under a C-arm X-ray fluoroscopy instrument (control group). The guide plate group comprised 7 male and 13 female patients, and the control group comprised 9 male and 11 female patients. Additional information about the patients is provided in Table 1. Patients were excluded if they presented with a pelvic, intertrochanteric, or open fracture. All patients were treated with the same type of cannulated compression screw implant by the same surgical team.

### 2.2. Digital 3D Model Reconstruction

The anatomical data of the patients were obtained by computed tomography (CT) scanning, and 3D models of the upper femur were reconstructed on the screen using medical digital imaging and an E3D digital medical modeling and design system (http://www.e3d-med.com; E3D Digital Medical and Virtual Reality Research Center, Central South University, Hunan Province, China). The surgeon then used a preoperative planning procedure to design the model. The surgeon chose an appropriate size for the required parts and recorded them. The model was stored as a stereolithography file, which was then sent to a 3D printer. The material of the 3D printing guide is polylactic acid (PLA).

### 2.3. Operation and Postoperative Follow-Up

Before the operation, the 3D-printed guide plate underwent plasma sterilization. After the femoral neck fracture was reduced by traction, the guide plate was placed percutaneously on the matching area of the proximal femur. With an assistant keeping the fracture stable, the surgeon maintained the position and direction of the guide plate stably with one hand and inserted the guide needle at the designed depth with the other hand. C-arm X-ray fluoroscopy was used to confirm that the insertion direction and guide needle depth were correct. Three cannulated compression screws were then implanted along the guide needle, according to the preoperative design. The position and angle of the three cannulated compression screws were determined by C-arm X-ray fluoroscopy, and the results were compared with the preoperative plan. The patients were followed up every 3 months. All patients were followed up successfully.

### 2.4. Statistical Analysis

Data were analyzed using Prism 7.0 statistical software (GraphPad, San Diego, CA, USA). Normally distributed data are expressed as the mean ± standard deviation and were analyzed using independent sample Student's *t*-test. Data that did not conform to a normal distribution were analyzed by the Mann-Whitney *U* test. *P* < 0.05 was considered to indicate a significant difference between the groups.

## 3. Results

### 3.1. Preoperative Data Acquisition, 3D Simulation Reconstruction, Screw Path, and 3D-Printed Guide Plate Design

**Figure 1:**
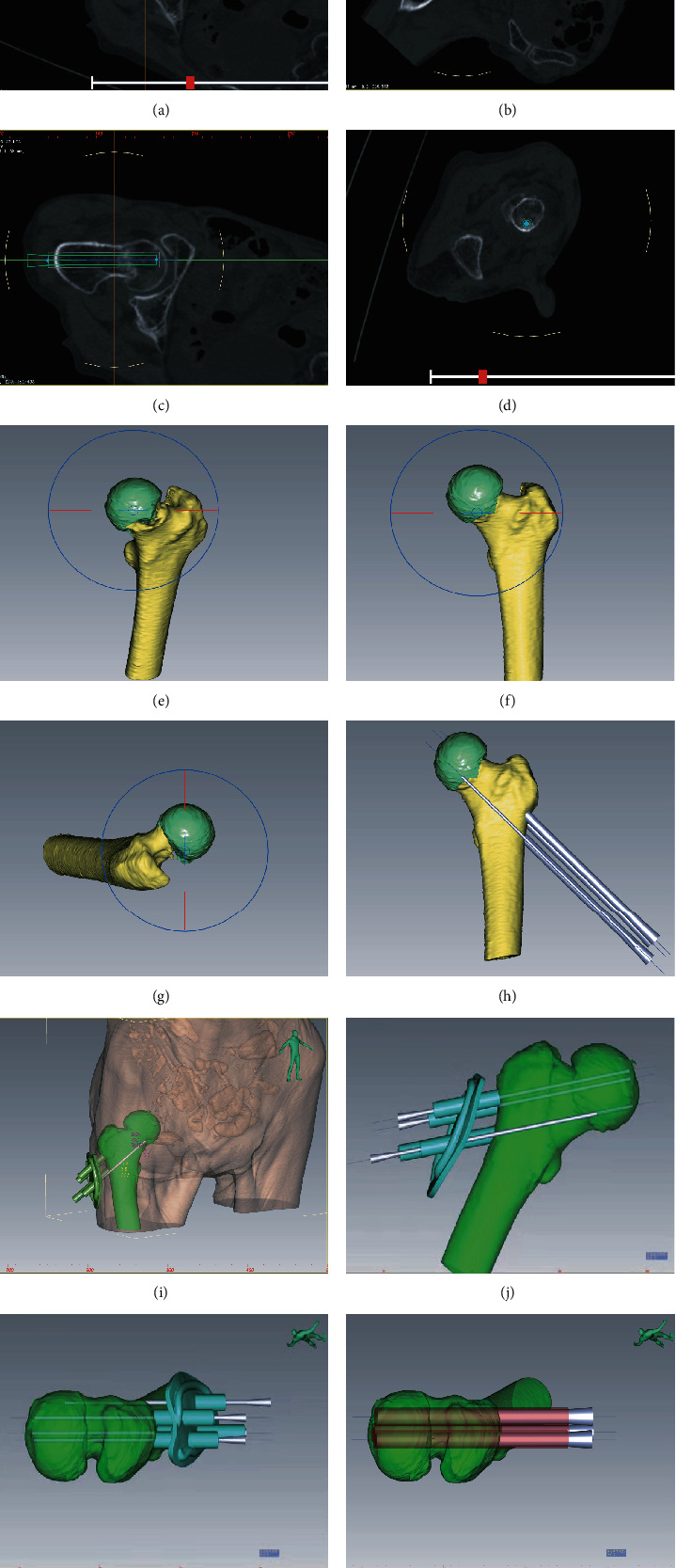
Digital 3D model reconstruction and preoperative planning. (a–d) CT scanning, multiplanar measurements, and reconstruction were performed for femoral neck fractures. (e–h) Computer-aided design and 3D simulation of femoral neck fracture reconstruction. (i–l) Computer-aided design and 3D simulation of the use of a guide plate and the surgical process of inserting three cannulated compression screws.

First, we performed a CT scan of the femoral neck fracture to collect detailed data (Figures 1(a)–1(d)). We then successfully simulated the reduction process of the femoral neck fracture using a computer, and fracture reduction was found to be satisfactory (Figures 1(e)–1(h)). Finally, we correctly evaluated the direction and length of the screw. The design of the 3D-printed guide plate was based on the anatomical characteristics of each individual (Figures 1(i)–1(l)).

### 3.2. The Accuracy of Computer-Aided Design and 3D-Printed Guide Technology

**Figure 2:**
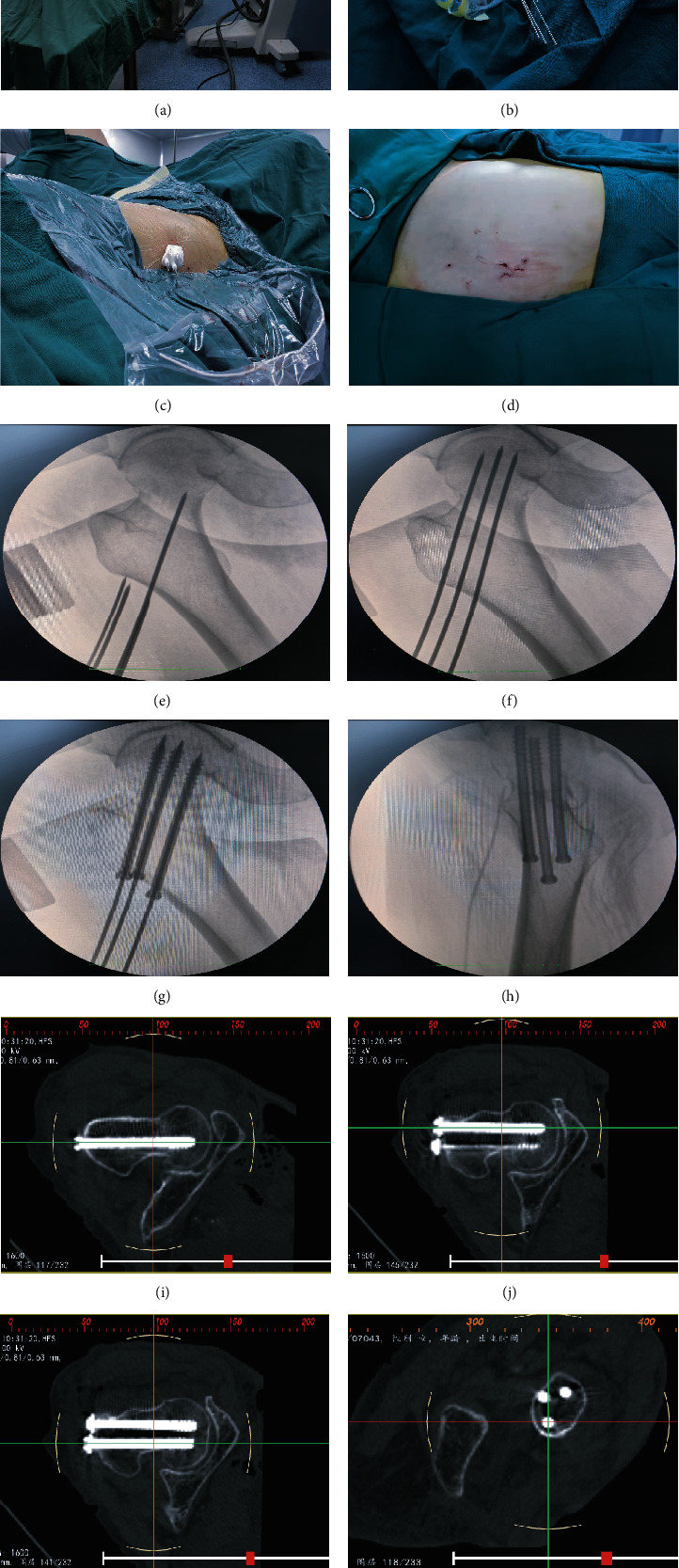
The accuracy of computer-aided design and 3D printing technology for guide plate production. (a–d) The surgical procedure of 3D-printed guide plate-assisted percutaneous needle insertion and cannulated screw placement. (e–h) C-arm X-ray fluoroscopy was used to observe the cannulated compression screw placement. (i–l) Immediate postoperative CT was performed to evaluate fracture reduction and the position of the three cannulated compression screws.

The 3D-printed guide plate was consistent with the anatomical characteristics of each individual. Under the guidance of the navigation template, the percutaneous insertion of the guide needle and the intraoperative C-arm fluoroscopy results were consistent with the preoperative design (Figures 2(a)–2(h)). CT scans immediately after the procedure showed that the reduction of the femoral neck fracture was satisfactory and that the position of the three cannulated compression screws was accurate (Figures 2(i)–2(l)). There were 17 cases of anatomical reduction and 3 cases of functional reduction in the guide plate group and 15 cases of anatomical reduction and 5 cases of functional reduction in the control group. The time taken to complete the procedure was significantly less for the guide plate group (35.3 ± 2.1 min) than for the control group (68.1 ± 4.9 min, *P* < 0.05). The amount of intraoperative blood loss was 6.3 ± 2.8 mL in the guide plate group and 21.3 ± 7.1 mL in the control group (*P* < 0.05). The guide plate group required significantly fewer X-ray fluoroscopy images (9.1 ± 3.5) than the control group (20.9 ± 2.8, *P* < 0.05).

### 3.3. Postoperative Follow-Up Results

**Figure 3:**
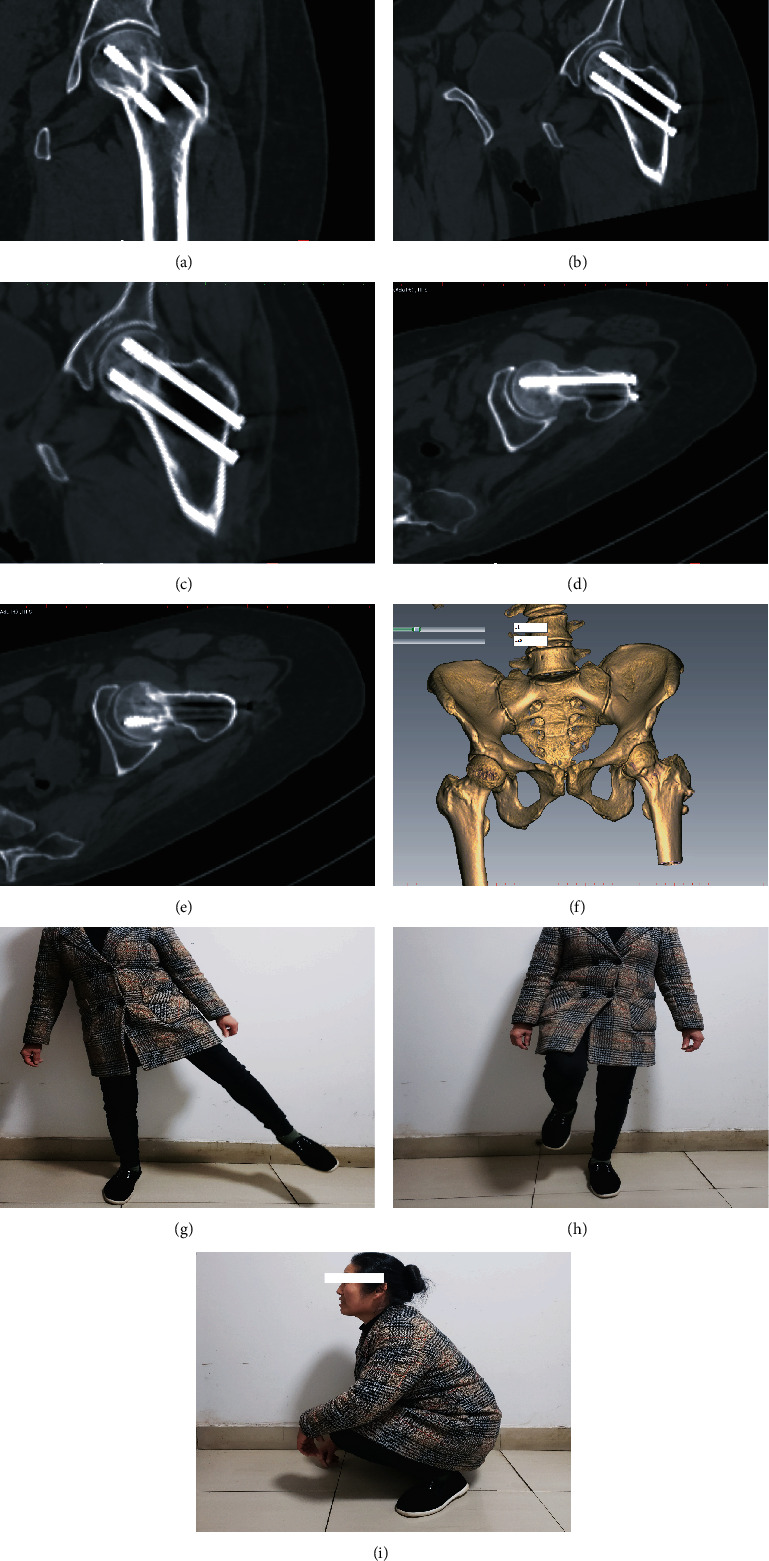
Postoperative follow-up results. (a–f) In the guide plate group, CT images show fracture healing in the final follow-up assessment of a representative 52-year-old female patient. (g–i) The hip function of a representative 52-year-old female patient at the final follow-up assessment.

All 40 patients were successfully followed up. The average follow-up time was 8 (range, 6–12) months. No avascular necrosis of the femoral head, screw withdrawal, or fractures occurred during the follow-up period (Figures 3(a)–3(f)). At the final follow-up assessment (Figures 3(g)–3(i)), the Harris score for hip joint function was 90.4 ± 3.5 in the guide plate group and 88.2 ± 2.8 in the control group (*P* > 0.05).

## 4. Discussion

The femoral neck fracture is the third most common type of fracture in traumatology, and various treatment options are available for it [1, 17]. Minimally invasive internal fixation with three cannulated compression screws is widely used to treat this type of fracture. The femoral neck shows good antishear force, antibending force, antirotation ability, and antiaxial stress after this fixation procedure. This procedure can reduce the incidence of nonunion of the femoral neck fracture and avascular necrosis of the femoral head. Moreover, the plane of screw placement is an inverted triangle and has the characteristics of triangular stability [18, 19]. Although this method has many advantages, there are certain challenges in its clinical application. First, it requires specialized surgical skills and extensive clinical experience, and it depends on intraoperative fluoroscopy to confirm that the screw position is accurate [20, 21]. Second, the screw placement process requires multiple fluoroscopy images, which increases the exposure of the patient and the surgeon to radiation. Third, intraoperative drilling and adjustment of the screw channel may affect the blood supply to the femoral head, thus reducing the holding force of the bone on the screw and consequently decreasing the stability of the fixation.

Therefore, a safe and effective method is needed to ensure the accuracy and safety of screw placement. A computer navigation system was once considered to be an acceptable auxiliary screw placement technology. However, the system's shortcomings, such as inconvenient operation, cumbersome registration, and high cost, prevented it from being widely used in clinical practice. With the development of digital medicine, 3D printing technology has become widely used in orthopedics. This technology allows the optimization of preoperative design and the realization of individualized and precise treatments. In addition, it has been reported that 3D-printed personalized guide plates are safe and feasible to use for the treatment of intertrochanteric fractures in adults and of hip diseases in children [16, 22].

In this study, digital orthopedic technology and 3D printing technology were combined in the guide plate group. A 3D-printed guide plate was used to assist in cannulated compression screw fixation of femoral neck fractures. Before the procedure, 3D reconstruction of the fracture site was performed using relevant software, and the resulting model could be rotated and transparently processed to preview the screw placement and adjust the screw path to the optimal path. Therefore, the required length and diameter of the screw could be determined before the procedure. A 1 : 1 fracture model was also printed before the procedure. The navigation template of auxiliary screw placement allowed the surgeon to simulate the procedure on the model, operate skillfully, and evaluate the difficulties and results of the procedure. It is also expected to be helpful for beginners to master the necessary surgical process and skills.

The use of a 3D-printed guide plate to assist the placement of cannulated compression screws for the internal fixation of femoral neck fractures has many advantages. First, it greatly simplifies and optimizes intraoperative procedures. The time taken to complete the procedure was greatly reduced, and the multiple intraoperative fluoroscopies, screw path sounding, and screw selection steps were eliminated. Specifically, the time taken to complete the procedure and the number of intraoperative X-ray fluoroscopy images were significantly less in the guide plate group than in the control group. Second, this procedure is convenient for junior doctors to master, and thus, they are more likely to promote this type of surgery. During the procedure, the guide plate only needs to be placed in the predesigned position, and the screw can be accurately placed without the need for multiple perspectives. Third, the biggest characteristic in this study is the positional Kirschner wire in front of the femoral neck and the designed catheter matching on the surface of the lateral femoral, thus guaranteeing the use of the minimally invasive method and the accuracy of the method at the same time. Moreover, because this guide plate-assisted procedure has a short learning curve and is easy to master, it can be performed in relatively poorly equipped hospitals.

## 5. Conclusion

In conclusion, the use of computer-aided design and a 3D-printed navigation template improved the accuracy of cannulated compression screw implantation for the treatment of femoral neck fractures. It reduced the risk of iatrogenic injury to the femoral neck and vascular system and reduced implantation time, intraoperative bleeding, and X-ray exposure. With further improvements in efficiency and safety, this technology has the potential to be widely adopted.

## Figures and Tables

**Table 1:** Patient information.

Group	Number of cases	Gender (M/F)	Age (years/range)	Injury side (right/left)	Garden classification (I, II, III, IV)
3D guide plate group	20	M: 7 F: 13	63.8 ± 16.9 50-93	R: 10 L: 10	6 II, 10 III, 4 IV
Control group	20	M: 9 F: 11	61.3 ± 19.8 45-85	R: 12 L: 8	8 II, 10 III, 2 IV

## Data Availability

The data used to support the findings of this study are available from the corresponding author upon request.
